# First description of the female of *Sarcophaga (Sarcorohdendorfia) gracilior* (Chen, 1975) (Diptera, Sarcophagidae)

**DOI:** 10.3897/zookeys.396.6752

**Published:** 2014-04-02

**Authors:** Ming Zhang, Jin-liang Chen, Xin-zhang Gao, Thomas Pape, Dong Zhang

**Affiliations:** 1 College of Nature Conservation, Beijing Forestry University, Beijing 100083, China; 2 Dalaoling Nature Reserve of Three Gorges in Yichang City, Yichang 443000, China; 3 Natural History Museum of Denmark, University of Copenhagen, Universitetsparken 15, DK–2100 Copenhagen, Denmark

**Keywords:** Sarcophagidae, *Sarcophaga (Sarcorohdendorfia) gracilior*, female, wing interference patterns, morphology, taxonomy

## Abstract

*Sarcophaga (Sarcorohdendorfia) gracilior* (Chen, 1975) is documented from specimens collected in Hubei Province, China, using morphological characters and wing interference patterns (WIPs). The female of *S. (S.) gracilior* is described for the first time, the male is redescribed, and both sexes are photographed. The distribution of the species is updated.

## Introduction

The Sarcophagidae (flesh flies) is a medium-sized family of Diptera with about 2600 known species worldwide, which includes various life history strategies ranging from inhabitants of pitcher plants to bat coprophages, crab saprophages, wasp nest inquilines, and insect parasitoids (Pape 1996). Some species are carrion breeders and therefore forensically important for the estimation of the time since death, i.e., the post-mortem interval (Greenberg 1991; Catts and Goff 1992; Amendt et al. 2004), and several species of these flies have been recorded in association with human remains (Sukontason et al. 2001, 2007; Chaiwong et al. 2009; Cherix et al. 2012).

*Sarcorohdendorfia* Baranov is a large subgenus of *Sarcophaga* Meigen (sensu lato), and it currently comprises 61 species known mainly from the Oriental and Australasian/Oceanian Regions (Pape 1996; Meiklejohn et al. 2013; Whitmore et al. 2013). The species *Sarcophaga (Sarcorohdendorfia) gracilior* (Chen, 1975) was originally described (in *Tricholioproctia* Baranov) based on eight male specimens from the type locality Mt. Tianmushan, Eastern China. Chen (1975) established the subgenus *Hamimembrana* with *Sarcophaga gracilior* as its type species and only member. Lopes and Kano (1979) treated *Tricholioproctia* as a junior synonym of *Sarcorohdendorfia*, and Pape (1996) considered *Sarcorohdendorfia* as a subgenus of *Sarcophaga* s.l., and listed the subgenus *Hamimembrana* as a synonym of *Sarcorohdendorfia*. Since its description, *Sarcophaga (Sarcorohdendorfia) gracilior* has remained unnoticed by the majority of the scientific community and has appeared in the literature mainly through brief citations and catalogue entries (Kano and Shinonaga 1994; Fan and Pape 1996; Pape 1996; Chen et al. 2010; Zhang et al. 2010). Besides, the morphology of the male had not been studied in detail and information on the female of this species was completely absent. During a distribution survey about flies of medical significance around Central China, we discovered three female specimens of *Sarcophaga (Sarcorohdendorfia) gracilior*, which to our knowledge represent the first record of reliably identified females. We herewith provide the first description of the female of *Sarcophaga (Sarcorohdendorfia) gracilior*, and a redescription of the male.

Wing interference patterns (WIPs) were recently introduced as a potential new character system of extremely thin insect wings (Shevtsova et al. 2011), and it has at this time proven useful for the separation of species in Hymenoptera, Hemiptera and Diptera (Buffington and Sandler 2011; Hansson 2011; Shevtsova and Hansson 2011, Shevtsova et al. 2011; Simon 2012). It might be suspected to provide a useful tool for correctly associating male and female specimens in some Sarcophagidae, and finds support in ongoing studies (Zhang et al., unpublished), and we therefore provide WIPs for both sexes of *Sarcophaga (Sarcorohdendorfia) gracilior*. This is the first time that WIPs are applied to a flesh fly.

The primary aims of this article are: 1) to provide the first description of the female of *Sarcophaga (Sarcorohdendorfia) gracilior* and a redescription of the male, and 2) to provide the first data on WIPs for flesh flies as a potential tool in associating conspecific males and females.

## Material and methods

Flies inhabiting forested areas in the mountainous region of the Hubei Province, China, were attracted by the viscera of grass carps (*Ctenopharyngodon idellus*) obtained from the local market. Viscera were kept frozen until needed, thawed and left to decompose for about two days before being deployed separately in traps consisting of open plastic containers (5.0 cm high, 10.0 cm in diameter). Flies that visited the bait during 1–2 hours from the time of deployment, were collected. Specimens were deposited in the Museum of Beijing Forestry University (MBFU), Beijing. Photographs were taken with a Canon 550D camera mounted on an Olympus SZX16 stereomicroscope. The methods applied to view and document interference colour patterns in the flies’ wings followed Shevtsova et al. (2011) and Shevtsova and Hansson (2011). Image processing softwares used were Adobe Photoshop CS3 (Adobe Systems, Inc., San Jose, CA, USA) and Helicon Focus 3.2 (Helicon Soft Ltd, Kharkov, Ukraine). Terminology of adult morphology follows McAlpine (1981). Distributional data was mainly taken from Pape (1996), with additional records obtained from major entomological catalogues (Chen et al. 2010; Zhang et al. 2010). The single male specimen was identified using Xue and Chao (1998) and by checking against the original description (Chen 1975). The female specimens were identified through careful comparisons with the male, supported by the fact that one pair (male + female) was collected *in copula*.

## Taxonomic account

### Sarcophaga (Sarcorohdendorfia) gracilior

(Chen, 1975)

http://species-id.net/wiki/Sarcophaga_gracilior

Tricholioproctia (Hamimembrana) gracilior Chen, 1975: 115. Type-locality: China, Zhejiang, Mt. Tianmushan.
Sarcorohdendorfia gracilior : Ye 1982: 22, 1992: 662; Fan and Pape 1996: 256; Xue and Chao 1998: 1646; Zhang et al. 2010: 360.
Sarcophaga (Sarcorohdendorfia) gracilior : Pape 1996: 397.

#### Female.

Description. Body length about 13.0 mm. Eyes bare. Fronto-orbital and parafacial plates black with golden yellow pollinosity, postocular strip black with silvery pollinosity; parafacial bristles in one row, fronto-orbital plate with rows of fine setulae. Frontal vitta black, about as broad as fronto-orbital plate at the narrowest point; frons at vertex 0.3 × head width; frontal row of 9–14 strong bristles; outer vertical bristle differentiated from postocular bristles, one reclinate and two proclinate orbital bristles. One pair of strong ocellar bristles, directed antero-laterally. Gena ground colour black, with black setulae in anterior 2/3, white setulae in posterior 1/3; height 0.3 × eye height in lateral view, postgena with white setulae. Antennal first flagellomere brown, not reaching the level of vibrissal insertion, 3.4 × as long as wide and 2.3 × as long as pedicel, pedicel black; arista long plumose in basal 2/3. Palpus black, expanded in distal part.

Thorax ground colour black, with yellow pollinosity; scutum with three black dorsal vittae. Chaetotaxy: acrostichals 5(6) + 1, dorsocentrals 4 + 4, intra-alars 1 + 2 (3), supra-alars 3 or 4, postpronotals 3, scutellum with 1 discal and 4 marginal bristles. Meropleurals 10 or 11, katepisternal bristles 1:1:1, prosternum, metasternum, proepisternum and postalar wall with dense black fine setulae. Wing hyaline; subcostal sclerite yellowish brown, bare; tegula black, with black setulae; basicosta light yellow, bare; costal spine not differentiated; vein R_1_ bare, three ventral setulae at node of R_4+5_-R_2+3_, vein R_4+5_ setulose dorsally from junction of R_2+3_ halfway to crossvein r-m; wing WIP (Fig. 5C) with clearly demarcated magenta and blue bands, and one large and almost triangular blue area on the apical part (shown with an arrow in Fig. 5C).

Legs dark, with grayish black pollinosity; fore femur with one row of dorsal bristles, one row of posteroventral bristles and one row of posterodorsal bristles, fore tibia with four anterodorsal and one posterior bristles; mid femur with four median anterior, one apical posterior and one apical posterodorsal bristles, mid tibia with two anterodorsal, one ventral and one subapical posterior bristles, and with one row of posterodorsal bristles (one strong); hind femur with one row of anterodorsal bristles, and with one apical posterodorsal and two apical posterior bristles, hind tibia with one row of anterodorsal bristles (among them three strong), and with one anteroventral and four posterodorsal bristles.

Abdomen oval with yellow pollinosity; tergite 3 without median marginal bristles, tergite 4 with one pair of median marginal bristles, tergite 5 with strong marginal bristles; sternite 2 with six long bristles along posterior margin. Terminalia: tergite 6 undivided (Fig. 2B), tergites 7+8 fused together (Figs 2C, 2E), sternite 2 with a small isolated sclerite on the posterior margin, sternites 5 and 6 rectangular in ventral view (Fig. 2A), sternite 8 represented by a membranous fold, hypoproct well developed but not particularly sclerotized and with numerous setulae (Fig. 2F), epiproct with only two strong bristles (Fig. 2E).

**Figure 1.**
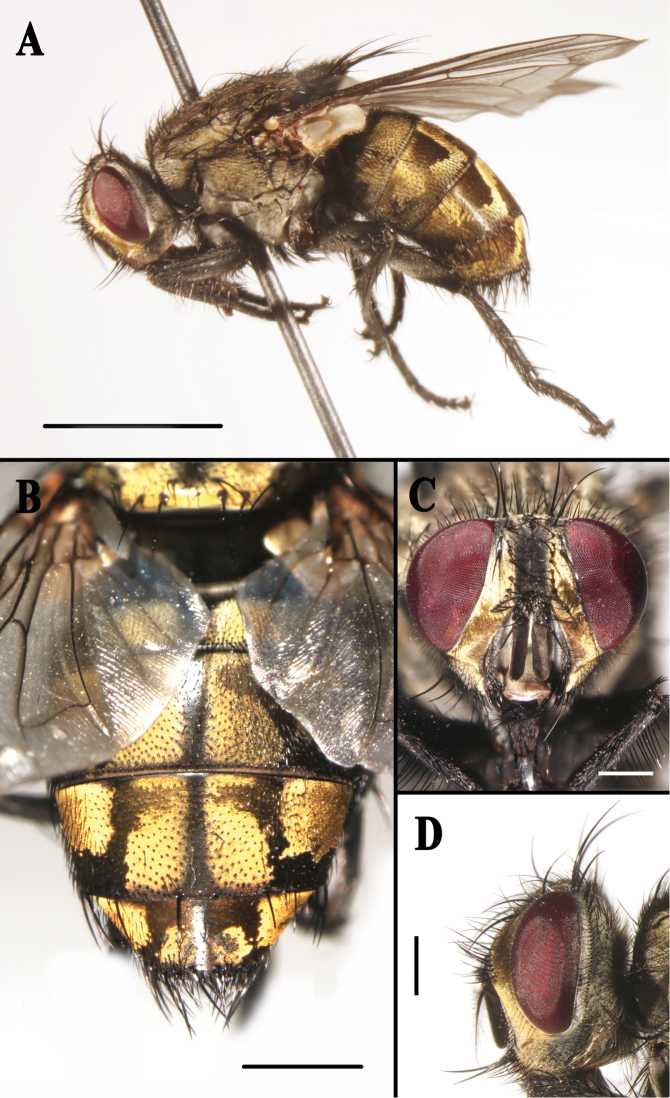
*Sarcophaga (Sarcorohdendorfia) gracilior* (Chen, 1975). Female. **A** Habitus, left lateral view **B** Abdomen, dorsal view **C** Head, anterior view **D** Head, left lateral view. Scale bars: **A** = 5.00 mm; **B** = 2.00 mm; **C** and **D** = 1.00 mm.

**Figure 2.**
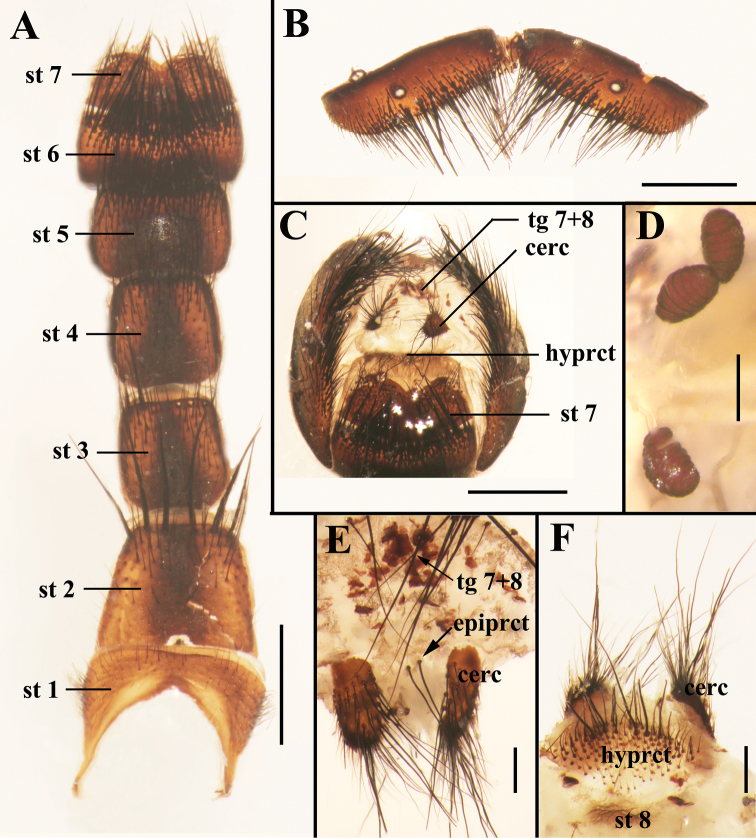
Photomicrographs of the female terminalia of *Sarcophaga (Sarcorohdendorfia) gracilior* (Chen, 1975). **A** Sternites 1−7, ventral view **B** Tergite 6, dorsal view **C** Terminalia, posterior view **D** Spermathecae **E** Terminalia, tergites 7+8, cerci and epiproct, dorsal view **F** Terminalia, cercus, hypoproct and sternite 8, ventral view. Scale bars: **A–C** = 1.00 mm; **D–F** = 0.25 mm. Abbreviations: cercus (cerc); epiproct (epiprct); hypoproct (hyprct); sternite (st); tergite (tg).

#### Male.

Redescription. Body length 16.0–17.0 mm. Frontal vitta 1.6 × as broad as fronto-orbital plate at the narrowest point; frons at vertex 0.22 × head width; frontal row of 11–13 bristles; outer vertical bristle not differentiated from postocular bristles, one reclinate orbital bristle. Antennal first flagellomere 4.1 × as long as wide and 3.1 × as long as pedicel.

Thorax: fore femur with slender ventral setulae in basal 1/2, fore tibia with three anterodorsal bristles; mid tibia with one anterodorsal bristle; hind femur with one row of anterior bristles, and with one apical posterior and three apical posterodorsal bristles, hind tibia with two posterodorsal bristles, and with slender and dense setulae along anteroventral and posteroventral surfaces.

Abdomen long oval; epandrium black; sternites 1−4 with dense setulae, sternite 4 with a dark spot consisting of dense short setulae on posterior margin (see Chen 1975: fig. 5). Terminalia (see Chen 1975: figs 6–9): cercus straight in profile, with numerous strong setulae on mid lateral margin and with a sharp apex in lateral and dorsal view. Surstylus almost triangular but with a slightly convex anterior (or ventral) margin (Fig. 4B). Pregonite with a broad base, slightly longer than postgonite, and distal half almost perpendicular to basal half, postgonite long triangular with slightly curved apex; vesica large; juxtal extensions small with a sharp tip in lateral view; lateral stylus slender, with recurving teeth in the distal half and situated under the arched juxta (Fig. 4A). Other morphological characteristics are the same as for the female.

**Figure 3.**
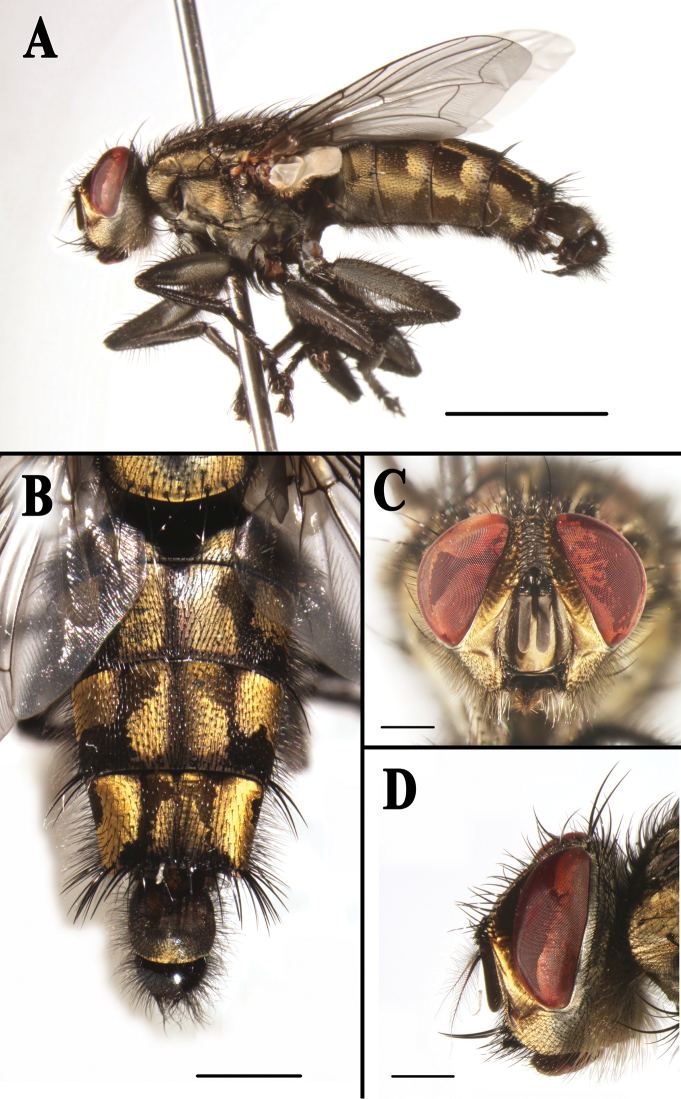
*Sarcophaga (Sarcorohdendorfia) gracilior* (Chen, 1975). Male. **A** Habitus, left lateral view **B** Abdomen, dorsal view **C** Head, anterior view **D** Head, left lateral view. Scale bars: **A** = 5.00 mm; **B** = 2.00 mm; **C** and **D** = 1.00 mm.

**Figure 4.**
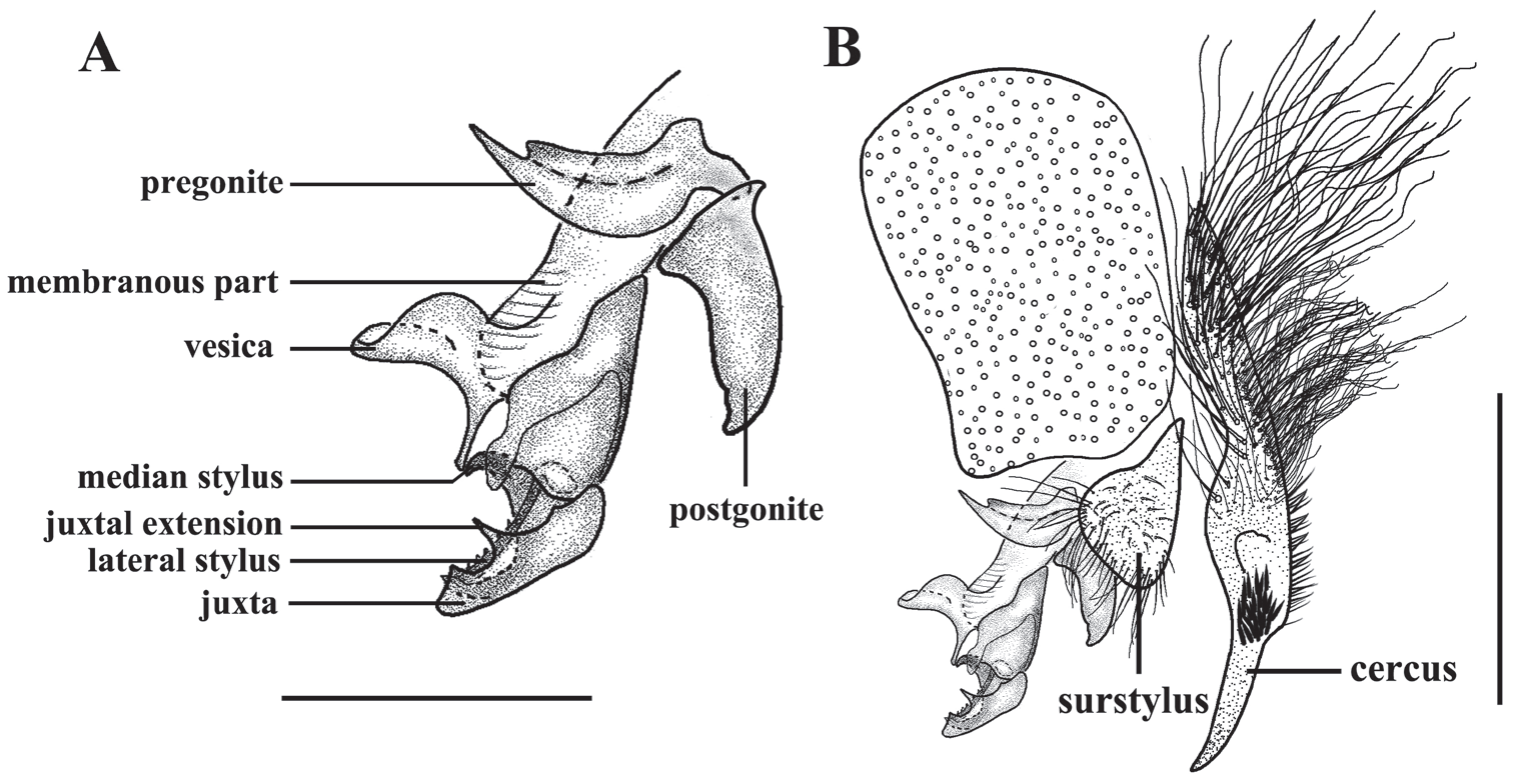
*Sarcophaga (Sarcorohdendorfia) gracilior* (Chen, 1975). Male. **A** Phallus and gonites, lateral view **B** Terminalia, lateral view. Scale bar: **A** = 0.50 mm; **B** = 1.00 mm.

**Figure 5.**
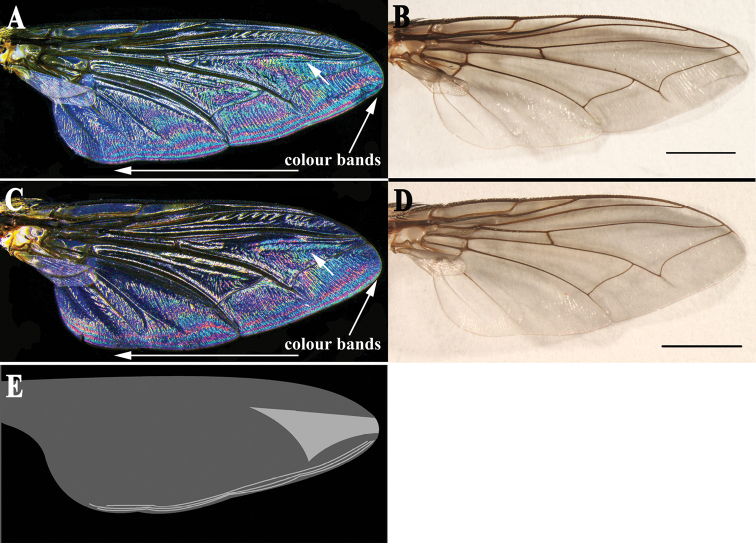
*Sarcophaga (Sarcorohdendorfia) gracilior* (Chen, 1975). **A** Male, right wing interference patterns, dorsal view **B** Male, right wing, dorsal view **C** Female, right wing interference patterns, dorsal view **D** Female, right wing, dorsal view. Scale bars = 2.00 mm **E** Schematic illustration of the distinctive clearly demarcated magenta and blue bands, and one large and almost triangular area on the apical part, which is blue in the WIP. Arrows in **A** & **C** show the most similar patterns and marginal colour bands of both sexes.

#### Material examined.

CHINA, Hubei, Yichang City, Dalaoling (31°5'00"N, 110°56'00"E): 1♀, Panlongling, 1600–1700 m, 17.VII.2013; 1♂, 1♀, Mt. Tianzhushan, 2000 m, 19.VII.2013; 1♀, Panlongling, 1600–1700 m, 22.VII.2013; all collected by Zhang D. & Zhang M.

#### Remarks.

The specimens of this species have been taken in traps baited with fish viscera, indicating that this species may be saprophagous like the majority members of the genus *Sarcophaga*.

#### Distribution.

China (Chongqing, Hubei [first record], Hunan, Guangdong, Guizhou, Sichuan, Taiwan, Xizang, Zhejiang), Nepal.

## Discussion

Females of most species of flesh flies are very similar in appearance and difficult to identify (Ye 1992; Pape 1996; Xue and Chao 1998), which represents a problem, e.g., for forensic investigators, because most specimens collected at death scenes are gravid females or larvae. Correct identification of females in the large genus *Sarcophaga* is very important, as it would be a prerequisite for many detailed ecological studies (e.g., Bänziger and Pape 2004; van der Niet et al. 2011), forensic investigations (e.g., Cherix et al. 2012), or cladistic analyses (e.g., Giroux et al. 2010). Morphological studies of *Sarcophaga* spp. have traditionally focused on the male sex, but Richet et al. (2011) and Meiklejohn et al. (2013) showed females are fully identifiable in many cases. To facilitate the identification of females in studies including *Sarcophaga (Sarcorohdendorfia) gracilior*, we provide the first description of the female and bring further distributional records of the species in China.

WIPs may arise in transparent insect wings due to their double layer of very thin cuticle (Shevtsova et al. 2011). The interference pattern caused by the ultra-thin but uneven wing membrane can be visualized against a dark background. WIPs have already proven to be of value in generic and even species-level identifications of several insect groups (Buffington and Sandler 2011; Hansson 2011; Shevtsova et al. 2011; Shevtsova and Hansson 2011; Simon 2012), and ongoing studies found WIPs to be species-specific and showing no sexual dimorphism in some taxa of Miltogramminae (Zhang et al. unpublished). We employed this method for comparisons between both sexes of *Sarcophaga (Sarcorohdendorfia) gracilior*, and the WIPs show no sexual dimorphism (Figs 5A, 5C). Studies of WIPs from other species of *Sarcophaga* are still needed to test if WIPs might serve as an appropriate way to confirm conspecificity of male and female specimens in the subfamily Sarcophaginae.

## Supplementary Material

XML Treatment for Sarcophaga (Sarcorohdendorfia) gracilior
